# Does Personality Predict Depression and Use of an Internet-Based Intervention for Depression among Adolescents?

**DOI:** 10.1155/2012/593068

**Published:** 2012-08-15

**Authors:** Hans Christian B. Vangberg, Kjersti R. Lillevoll, Knut Waterloo, Martin Eisemann

**Affiliations:** Department of Psychology, University of Tromsø, 9037 Tromsø, Norway

## Abstract

*Background*. Focus upon depression and prevention of its occurrence among adolescents is increasing. Novel ways of dealing with this serious problem have become available especially by means of internet-based prevention and treatment programs of depression and anxiety. The use of Internet-based intervention programs among adolescents has revealed some difficulties in implementation that need to be further elucidated. The aim of this study is to investigate the association between personality and adolescent depression and the characteristics of users of an Internet-based intervention program. *Method*. The Junior Temperament and Character Inventory (JTCI), the General Self-Efficacy scale (GSE) and the Centre for Epidemiological Studies-Depression scale (CES-D) have been administered to a sample (*n* = 1234) of Norwegian senior high-school students. *Results*. Multiple regression analysis revealed associations between depression and gender, and several JTCI domains and facets. In line with previous findings in adults, high Harm Avoidance and low Self-Directedness emerged as the strongest predictors of adolescent depressive symptoms. Further, in logistic regression analysis with the covariates JTCI, GSE and CES-D, the only significant variables predicting use/non-use were the CES-D and the temperament domain Reward Dependence. *Conclusion*. The results in this study revealed level of depressive symptoms as the strongest predictor of the use of the Internet based intervention and that personality might provide useful information about the users.

## 1. Introduction

The prevalence of depression in childhood is low, whereas adolescence is a period of life characterized by a substantial vulnerability to depression [1]. Accordingly, adolescent depression is highly prevalent [2], with a considerable risk of recurrence and is often followed by poor psychosocial functioning and scholastic outcome [3].

Factors affecting development and predisposition to depression are numerous [4]. Grant and colleagues [5] point to the importance of stressful negative life events in understanding the development of depression, while others [6] indicate the importance of genetic factors. Interpersonal vulnerabilities have also been investigated for significant factors that can contribute in the explanation of adolescent depression [4]. Cognitive vulnerability concerns mostly about how an individual perceives, interprets, and reasons about experiences and relationships. Cognitive factors receiving most attention are negative inferential styles [7], dysfunctional attitudes [8], tendencies towards rumination [9], and self-criticism [10]. Based on various vulnerabilities, Hankin [4] suggested a multifactorial approach to the study of depression.

There is increasing evidence that psychiatric disorders have specific associations with underlying temperament and character traits among children and adolescents [11–13]. The interrelationships between personality, depression and anxiety have been studied over the last decades in adult populations [14–22], and several personality traits have been identified affecting mood disorders, such as neuroticism [23–25], tendencies towards rumination [25], immature personality styles, and personality disorders [26, 27]. 

 A model to assess temperament and character to describe underlying biogenic structures of personality had been developed by Cloninger et al. [28] resulting in the Temperament Character Inventory (TCI) that measures personality by two higher-order dimensions: temperament and character. Temperament varies on an individual basis and reflects the fundamental organization of brain systems responsible for activating, maintaining and/or inhibiting behavior in response to stimuli [14, 28]. The four derived temperament dimensions are *novelty seeking (NS), harm avoidance (HA), reward dependence (RD),* and *persistence (P)*. On the other hand, character refers to self-concepts and individual differences in goals and values, which in turn affect voluntary choices and intentions. According to Cloninger [29], character is moderately influenced by sociocultural learning and matures gradually throughout life. The three described character dimensions are *self-directedness (SD), cooperativeness (C),* and *self-transcendence (ST)*. These aspects of personality interact to allow for adaptation to life experiences and also influence the vulnerability for emotional and behavioral disorders. 

Scores on the TCI have shown to be variant between depressed individuals and nondepressed [28]. Elevated scores on HA and lower scores on SD and C have often been found associated with depression or depressed mood in adult populations [30–32]. 

Since the adult version of the TCI might be unsuitable for use in an adolescent population, Luby et al. [33] developed the Junior Temperament and Character Inventory (JTCI).

 So far, research on the relationship between JTCI and depression among adolescents is sparse. A few studies have reported associations between JTCI and psychopathology in adolescents [34–38]. Schmeck and colleagues [34] reported a negative correlation between several forms of psychopathology, including anxiety and depression and SD. Others [37] have found high scores on NS and HA, while low on RD and P among dysregulated children. Elevated levels of HA have also been reported among depressed adolescents [36].

 Different relationships between temperament and character traits and mental health are suggested to exist in adolescents and adults [39]. Therefore, more research is needed to improve our understanding of the relationship between adolescent depression and personality. 

One might expect that many depressed adolescent individuals will never formally seek help or contact with health services [40], rendering many with an unmet need for treatment [41]. This is a strong argument for finding novel approaches to reach adolescents struggling with mental health problems. Internet-based interventions like computerized cognitive behavior therapy (cCBT) is one way to reach a broader spectrum of troubled youth. One of these interventions is the MoodGYM, which is a self-help program developed at the Australian National University based on the principles of cognitive behavioral therapy (CBT) [42–44]. It consists of a set of five training modules aimed at increasing the users' knowledge about their symptoms, negative automatic thoughts, dysfunctional attitudes, emotions, and coping strategies with regard to stress and interpersonal relationships. Furthermore, the MoodGYM comprises a personal workbook that records and updates the user's responses and a feedback evaluation form. 

The use of internet-based interventions among adolescent samples has revealed some difficulties in implementation [45]. Motivating young people to seek help for mental health problems also emerged as a challenge. One reason for low adherence might be that cCBT applications allow the users to enter and leave a treatment program and thus resulting in a lack of commitment to the treatment. Determining those using and benefiting from Internet interventions is an important issue. The identification of their characteristics could be useful for increasing adherence and for the prediction of treatment outcome [46–48]. Several hypotheses about possible relationship between different JTCI domains the use of MoodGYM can be formulated. It can be expected that high scores on SD and CO, and low scores on RD predict more use due to the nature of these domains [28]. It is reasonable to assume that a high score on SD to some degree reflects the internal drive and goal-orientation necessary to engage in a self-directed program such as MoodGYM. High scorers on CO might be more motivated to fulfill their commitment as participants in a research project and therefore be more likely to make use of the intervention. Individuals scoring low on RD are characterized as practical, socially distant, and independent. They might find this sort of intervention appealing because of the practical, socially independent nature of Internet-based interventions. 

The concept of self-efficacy refers to a person's “conviction, that one can successfully execute the behavior required to produce the outcomes” (Bandura, 1977, p. 193 [49]) and thus is related to motivation and behavior. Self-efficacy has been found to be a significant predictor of motivation for learning-directed behavior [50]. With regards to help-seeking behavior in adolescents, Barker et al. [51] claim that research evidence on the influence of self-efficacy is conflicting. Nonetheless, it might represent a factor promoting help-seeking behavior and affecting the intervention outcome in mental health prevention programs. Contrary to their hypothesis, Pössel et al. [52] found students low on self-efficacy to benefit more from a depression prevention program. The current study will investigate whether self-efficacy can predict self-directed use of MoodGYM when presented to students in a school-based setting.

Being able to depict the role of personality and adolescent depression in this context it is important to understand fundamental and underlying mechanisms in order to get a better understanding and treatment of it. If researchers can understand what underlies the development and vulnerability towards depression among youth, treatment can be modified to better target key factors. Internet-based interventions for depression are, compared to traditional face-to-face treatment, easy to present and to implement.

## 2. Aims

The aims of the current study are twofold: firstly, to assess the predictive power of gender, age, and personality aspects (Junior Temperament Character Inventory, JTCI) for the severity of depressive symptoms (Centre for Epidemiologic Studies Depression scale, CES-D), and secondly, to explore the characteristics of users versus nonusers of an Internet-based intervention program (MoodGYM) using the variables JTCI (domain and facets), GSE, gender, and CES-D.

## 3. Method

### 3.1. Procedure

Participants were recruited from four senior high schools in Troms County in Northern Norway as a part of a larger study with a four-arm randomized controlled trial design with repeated measures (pre- and post- intervention). Members of our research group visited the four participating schools for recruiting the participants. The recruitment process involved the delivery of a short informative lecture about mental health in general and a presentation of the MoodGYM program, followed by an invitation to the students to participate in the study. Students volunteering to participate signed a written consent form. If the students did not want to participate in the MoodGYM trial, they had the choice of participating in the preintervention survey only. As an incentive for becoming a participant, a lottery drawing was announced. There was an additional lottery for those participating in the MoodGYM trial.

The data collection for the current study was part of the preintervention survey, which was conducted by computer in a classroom setting on the day of the information session. The students without access to a computer completed a paper version. 

Students willing to participate in the MoodGYM trial received username and passwords on e-mail within a week of recruitment for registration in the program. The use of MoodGYM was self-directed, without personal followup, and no time was allocated during school hours. Within 6–8 weeks, the research team returned to collect postintervention data.

The regional medical research ethics committee approved the study. Data of use of MoodGYM were securely stored on a server at the Australian National University and were retrieved for information about use/nonuse for participants in the trial.

### 3.2. MoodGYM

MoodGYM is an internet-based self-help program based on the principles of cognitive behavior therapy (CBT). It has been developed at the Centre for Mental Health Research at the Australian National University in Canberra. The aim of this program is prevention and treatment of depression by means of five modules and a personal workbook [53]. Each module has a specific theme and is designed for increasing the users' knowledge about their own symptoms, negative automatic thoughts, dysfunctional attitudes, emotions, and coping strategies with regard to stress and interpersonal relationships. Each module takes between 30–45 minutes to be completed.

## 4. Outcome Measures

### 4.1. Depressive Symptoms

Level of depression was measured using a Norwegian version of the Centre for Epidemiologic Studies-Depression scale (CES-D) [54], developed to measure depressive symptomatology in the general population. This 20-item self-report scale yields scores ranging from 0 to 60 (scores given from 0 to 3), with a score of 16 or above indicating a clinical level of depression. However, we used a cutoff score above 24 was used which is assumed to detect more accurately clinically cases among adolescents [55, 56]. The CES-Ds Cronbach alpha in the current study was .884.

### 4.2. Self-Efficacy (GSE)

Self-efficacy was measured using the Norwegian version of the General Self-Efficacy Scale (GSE) [57]. The scale consists of 10 items and assesses the ability of an individual's beliefs in handling difficult situations in an appropriate way. Responses are reported on a four-point scale ranging from “not at all true” to “exactly true.” Its Cronbach alpha in the current study was .882.

### 4.3. Junior Temperament and Character Inventory (JTCI)

The JTCI is a self-administered questionnaire containing 106 items scored on a five-point scale (1 to 5) ranging from “totally agree” to “totally disagree.” The Norwegian version of the JTCI was developed according to established guidelines following several steps based on the German version of the JTCI [58]. This procedure included translation, backtranslation by independent native speakers, and linguistic revision of items [59]. 

 The Cronbach alpha for the current study were for NS = .795, HA = .846, RD = .792, *P* = .793, SD = .846, CO = .803, and for ST = .796. 

### 4.4. Demographics

Demographic parameters were gender, age, and grade in high school.

### 4.5. Statistical Analysis

All statistical analyses were performed with the SPSS version 18 and 19 for Macintosh. To identify unique JTCI correlates and predictors of depressed mood, hierarchical regression methods were used. For the domain-level analysis, all JTCI domains were subjected to forced entry in the same analytic step and their unique contributions simultaneously evaluated. In the facet-level analysis all of the 29 facets scales were entered in the same model using forward entry.

Bonferroni adjustment was applied to the critical *α* level and for significance tests involving the seven domains and the 29 facet scores on the JTCI. This adjustment resulted in a critical *α* level of .0071 (.05/7) and .0017 (.05/29), respectively.

Direct logistic regression was run to assess the impact of nine independent variables (CES-D, GSE, and the JTCI subscales) on the likelihood that participants in the study would use MoodGYM or not.

Another direct logistic regression assessed in more detail the impact of JTCI facets on users versus nonusers of MoodGYM. The model contained thirty-two independent variables (CES-D, GSE, gender, and the twenty-nine JTCI facets).

## 5. Results

This study was conducted on adolescents from Norwegian senior high schools. The sample comprised 604 males (48.7%) and 635 females (51.3%) with a mean age of 16.8 (range = 15–20). Women scored significantly higher on CES-D than men *F*(1,1233) = 84.32, *P* < .001 with a mean of 15.78 and 11.00, respectively (Table 1). The total mean score for CES-D in this sample was 13.45. The percentage scoring above the cut-off of 16 was 30.7%, whereas the percentage above the cut-off of 24 was 14.3%.

When CES-D scores were correlated with the seven JTCI domains all coefficients emerged as significant (Table 2). HA (*r* = .56, *P* < .01) and SD (*r* = −.64, *P* < .01) yielded the strongest association with depressed mood. Somewhat lower significant associations were obtained for *P*(*r* = −.34, *P* < .01), while small ones emerged with NS (*r* = .09, *P* < .01), RD (*r* = −.14, *P* < .01), CO (*r* = −.12, *P* < .01), and ST (*r* = .16, *P* < .01). 

### 5.1. Regression

A hierarchical multiple regression analysis was applied to assess the power of gender, age and personality to predict depressive symptoms (CES-D). A preliminary analysis was conducted to disclose any violations of assumptions. Gender and age were entered into step one, explaining 6.5% of the variance in depression (Table 3). After the entry of the JTCI at step two, the total variance explained totaled to 46.3%, *F*(9,1221) = 118.88, *P* < .001. The JTCI explained an additional 40.2% of the variance in depression, *R* square change = .402, *F* change (7,1221) = 131.42, *P* < .001. In the final model the following measures were statistically significant: gender (*P* < .001, beta = .144), and after Bonferroni correction: NS (*P* < .001, beta = .109), HA (*P* < .001, beta = .181), RD (*P* < .001, beta = −.089), SD (*P* < .001, beta = −.487), and CO (*P* < .001, beta = .110).

### 5.2. Facet-Level Analyses

A more detailed analysis was performed on facet level of the JTCI domains to check for their ability to predict depression. For this analysis, facet scale scores were entered into the hierarchical regression analysis at the second step in the model using forward selection after age and gender were forcedly entered in step one. Nine of the 29 facet scales emerged as unique and significant predictors. Together with gender and age, they explained 52.8% of the variance in CES-D scores (Table 4). Gender was found significant (*P* < .001). Significant facets, after Bonferroni correction, were SD4 (self-striving) (beta = .24, *t* = −8.06, *P* < .001), SD2 (lack of goal direction) (beta = −.277, *t* = −10.72, *P* < .001), HA1 (anticipatory worry) (beta = .16, *t* = 5.56, *P* < .001), and RD4 (independence) (beta = −.80, *t* = −3.76, *P* < .001).

### 5.3. Logistic Regression of the JTCI Domains

Direct logistic regression was performed to assess the impact of a number of factors on the likelihood that participants in the study would use MoodGYM or not. The number of users in this sample was not optimal, with only 51 (7.3%) participants actually logging in and using the MoodGYM program.

The model contained ten independent variables (CES-D, GSE, gender, and the JTCI subscales). The overall model was significant *χ* ^2^(10, *n* = 691) = 20.71, *P* < .05, indicating that the model was able to distinguish between participants that used and those who did not use MoodGYM. The model as a whole explained between 3.2 (Cox and Snell *R* square) and 7.9% (Nagelkerke *R* square) of the variance in use/nonuse and correctly classified 45.2% (an improvement of 37.8% from the model where only the constant is included) of the cases. Further, the Hosmer and Lemeshow test has a significance level of .584, thus indicating that the model prediction does not significantly differ from the observed. The Wald criterion showed (Table 5) that only two of the independent variables made a unique statistically significant contribution to the model (CES-D and the subscale RD on the JTCI). Gender, GSE, and six of the JTCI domains were not significant predictors. The strongest predictor of use/nonuse was CES-D, yielding an odds ratio of 1.05 (*P* < .05), and indicated that those who reported a higher level on CES-D were more likely to use MoodGYM than those who scored lower on CES-D, controlling for all the other factors in the model. This means that for every point a participant increased on the CES-D score, the odds of using MoodGYM increased with 5% (O.R. = 1.05). The odds ratio of .953 for RD (reward dependence) indicates that for every additional point on RD the odds were 4.7% less for the participants to use MoodGYM, controlling for other factors in the model. 

### 5.4. Logistic Regression on the JTCI Facets

To investigate in more detail the traits of MoodGYM users, a direct logistic regression was performed to assess the impact of the JTCI facets on the likelihood that participants in the study would use MoodGYM or not.

The model contained thirty-two independent variables (CES-D, GSE, gender, and the twenty-nine facets of the JTCI). The overall model was significant *χ* ^2^(31, *n* = 691) = 59.92, *P* ≤ .001, indicating its ability to distinguish between participants who used versus not used MoodGYM. The model as a whole explained between 8.3 (Cox and Snell *R* square) and 20.3% (Nagelkerke *R* square) of the variance in use/nonuse and correctly classified 60.9% (an improvement of 53.5% from the model where only the constant is included) of the cases. Furthermore, the Hosmer and Lemeshow test yielded a significance level of .241, thus indicating that the model prediction does not significantly differ from the observed. The Wald criterion demonstrated (Table 6) that seven of the independent variables made a unique statistically significant contribution to the model (CES-D, gender, the JTCI facets ns4, ha1, ha4, rd4, p4, and sd3). The strongest predictor of use/nonuse was gender, yielding an odds ratio of 2.65 (*P* < .05) indicating that female students have a 26.5% increase of odds for using MoodGYM. Findings are reported in Table 6.

## 6. Discussion

The findings from this study underline the potential importance of personality in adolescent depression. The JTCI factors harm avoidance and self-directedness, yielded the strongest associations. In the regression model, JTCI domains explained additional 40.2% of the variance in CES-D after having controlled for age and gender. NS, HA, RD, SD, and CO emerged as significant correlates in explaining depression among adolescents, which is slightly different from an adult population [15, 16, 23]. Cloninger et al. [28] have described individuals with high scores on the temperament domain NS as exploratory and curious, impulsive and disordered while high scores on HA are associated with being worried and pessimistic, fearful, shy, and fatigable. Further, low scores on RD reflect a cold personality, detached and independent from others. Another association was found between CES-D and the character domains of SD and CO. Scoring low on SD is found to be associated with being immature and fragile, blaming, unreliable, ineffective, and having problems working towards long-term goals. Individuals scoring high on CO are described as socially tolerant, empathic, compassionate, and ethical. Studies report a significant negative correlation between depression and CO [15, 16, 23], which is confirmed in the current study. However, when included in the final regression model and considered together with other domains the negative correlation between CES-D and CO turns into a positive beta coefficient. This suppression effect seems to be due to variations of positive and negative correlations between the other personality domains and CES-D, but leaving the true direction of the association between CO and CES-D for this sample in a positive direction. As shown above, the strongest correlations toward CES-D occurred between SD and HA, which is in line with previous findings [15, 23]. Cloninger et al. [15] found that HA functions as a marker for vulnerability to depression, while SD was a marker of a central protective function towards depression. The current findings exhibit depressed individuals as anxiety-prone and immature. The reason for the positive relationship between CO and depression might be due to being very empathic and considerate, thus experiencing an emotionally oversensitivity, leading to lowered mood states. 

The facet level analysis reveals a more detailed picture of traits affecting depressed mood. The second regression model shows that the JTCI facets explain 46.4% of the variance in CES-D. The following facets emerged as significant associations with CES-D: SD4 (self-striving), SD2 (lack of goal direction), HA1 (anticipatory worry), and RD4 (independence). Cloninger et al. [28] described individuals low on SD4 as self-striving people, never accepting nor enjoying their actual mental and physical features. They also have severe problems adjusting perceptions of themselves when corrected by the environment. High scores on SD2 characterize persons having difficulties in finding direction and meaning in their lives and instead react to current situations and immediate needs. Scoring high in HA1 indicates two types of behavioral tendencies. Such individuals are pessimistic, expecting failure and harm, especially in adverse and unfamiliar situations. Further, these individuals have difficulties in forgetting embarrassment and a tendency to ruminate about stressful situations for long periods of time. And finally, scoring low on RD4 describes individuals who are independent and accordingly do not seek emotional support and approval from the environment. They appear insensitive to social pressure, criticism and rarely give in to the wishes of others. 

The present study was conducted among a younger sample that might affect which domains and facets emerge as specific. The results from the facet level analysis reflect adolescents with elevated depressive symptoms as individuals lacking the ability to work towards long-term goals, which in addition, worry and expect the worse in situations even when there is support and assurance available. They resemble withdrawn, independent individuals, who apparently are insensitive to social pressure, which at first glance might display them with a strong personality, not vulnerable to depression. This finding might be a combined effect of the other facets and seems like a natural response when individuals struggle with their self-image at the same time as they worry and expect negative outcomes and do not cope adequately with embarrassment. A subject with such a personality will most likely not express true feelings. 

The challenges concerning adherence of online CBT users deserve further investigations [46, 47]. Describing the users of Internet interventions will probably increase the ability to predict adherence and intervention outcome. The results from the logistic regression used in the current study revealed that the only predictors for the use of MoodGYM were depression and RD (reward dependence). This is both in accordance with findings of some studies [47] but also contrasts others [48]. The indication that higher scores on CES-D predictive use of this online intervention tells us that presenting this kind of intervention to the population will most likely facilitate use among individuals with elevated symptoms on depression. This highlights the importance of finding effective strategies to maintain adherence among those who decide to use the program. Another significant predictor was reward dependence. Cloninger et al. [28] described individuals scoring low on this temperament trait as cold, socially insensitive, preferring to keep distance to others, and having objective views that are not romanticized by a desire to please others or wishful thinking. A possible explanation for this domain being a significant predictor for use might be that these individuals prefer a self-help approach to deal with their problems, rather than seek help from others. 

 In order to give a more detailed description of the users of MoodGYM, a second logistic regression was done including the same predictors, but based on the JTCI facets. Here, gender was the strongest predictor, indicating that female students are more likely than male students to use this kind of intervention. The level of depressive symptomatology also predicted its use, reflecting that depressed individuals might actively use this kind of program. The facet sd3 (resourcefulness) also emerged as a strong predictor of use, indicating that the users are resourceful and effective individuals, focused on solving problems and are perceived by others as competent and productive. On the other hand, they score lower on p4 (pragmatic) [29], describing them as somewhat lazy underachieving individuals who can be seen as pragmatics accepting compromises easily [28]. Further, they obtain low scores on rd4 (independence) implying that they rarely seek emotional support from others, rather impress others as self-sufficient and they are not reacting to social pressure. Another significant facet, ha1 (anticipatory worry) describes individuals as worried and anticipating harm. Ha4 (vigor) also emerged as specific. Low scores on this facet depict individuals as energetic and dynamic, with less need for rest, who rarely have to push themselves. Further, ns4 (regimentation) emerged significant in predicting use, identifying individuals who tend to be organized, methodical, and systematic. They prefer activities with strict rules and are able to postpone gratification longer than most of us. 

This gives a picture of the MoodGYM users as effective and strict rule-followers, working effectively in order to achieve goals, at the same time as they seem similar to independent individuals reluctant to take into account what others do or mean. These individuals' worry of failure might function as worry of failure might function as a facilitator towards goal-directed behavior, at the same time as it reflects their vulnerability to depression. They also seem to be like resourceful individuals who are underachieving, holding a pragmatic view upon what they are doing. These aspects, together with the level of depression symptoms and gender, moderately predict use of MoodGYM, indicating that there could exist other important factors that will better predict its use. 

The assessment of the characteristics of internet-based intervention users is an intriguing aspect of online delivery healthcare research that needs further investigation. The fact that only a few students used the MoodGYM program might indicate that this kind of intervention is most appealing to and effective in individuals with specific characteristics. The delineated characteristics of MoodGYM users might aid us in the further development of internet-based programs, their implementation and last but not least facilitate uptake and adherence. 

## 7. Limitations

Data collection occurred in a classroom environment, not ensuring sufficient privacy for the participants. Further, the effect sizes reported are small. Also the variables failed to explain a larger portion of the variance, indicating that definite conclusions of both predicting use/nonuse and depression cannot be drawn. A relatively small number of MoodGYM users (7.3%) also compromise the study. Further, the large sample size also raises concern about unreliable responses. To minimize the possible effect, repeated “data-washing” was made, and this effect is assumed to be minimal. Finally, this group of adolescents had not been specifically asked to take part in a treatment intervention, which may also affect the outcome and possibly the low rate of adherence to the intervention. Since this investigation represents part of a larger study on identifying and exploring different aspects of MoodGYM after its introduction in Norway, its results should be regarded as preliminary.

## Figures and Tables

**Table 1:** Mean scores and standard deviations for male and female (*n* = 1239).

	Male (*n* = 604)		Female (*n* = 635)	
	$\overset{-}{x}$	SD	$\overset{-}{x}$	SD
CES-D	10.996	8.334	15.776	9.845
GSE	29.842	5.119	29.001	4.234
NS	47.200	8.433	46.106	7.877
HA	36.480	8.473	40.962	8.832
RD	56.551	9.201	60.243	9.092
P	47.124	7.500	45.847	7.591
SD	51.940	9.408	49.230	9.341
CO	62.810	8.721	65.984	7.984
ST	26.204	7.098	28.224	7.256

CES-D: Centre for Epidemiological studies-Depression Scale; JTCI: The Junior Temperament and Character Inventory; NS: novelty seeking; HA: harm avoidance; RD: reward dependence; P: persistence; SD: self-directedness; CO: cooperativeness; ST: self-transcendence.

**Table 2:** Correlation table of the variables in the hierarchical multiple regression (*n* = 1231).

	CES-D	Age	NS	HA	RD	P	SD	CO	ST
CES-D	—	.026	.093**	.559**	−.140**	−.339**	−.642**	−.115**	.155**
Age		—	−.037*	−.007	−.026	.018	.025	.079**	−.031
NS			—	−.059*	.216**	−.210**	−.109**	−.253**	.208**
HA				—	−.178**	−.427**	−.711**	−.153**	.261**
RD					—	.204**	.231**	.450**	.182**
P						—	.494**	.478**	−.001
SD							—	.305**	−.183**
CO								—	.111**
ST									—

CES-D: Center for Epidemiological studies-Depression Scale; JTCI: The Junior Temperament and Character Inventory; NS: novelty seeking; HA: harm avoidance; RD: reward dependence; P: persistence; SD: self-directedness; CO: cooperativeness; ST: self-transcendence. **P < .01, *P < .05.

**Table 3:** Hierarchical multiple regression analysis of CES-D as a function of JTCI domain scales after controlling for gender and age (*n* = 1230).

Correlates	Standardized beta coefficients	
	Model 1	Model 2
Gender	.254^∗∗∗^	.144^∗∗∗^
Age	.032	.036
NS		.109^∗∗∗∗^
HA		.181^∗∗∗∗^
RD		−.089^∗∗∗∗^
P		−.023
SD		−.487^∗∗∗∗^
CO		.110^∗∗∗∗^
ST		−.017
_Adj_ *R* ^2^	.064	.463
Δ*F*	42.76^∗^	131.42^∗^
Δ*R* ^2^	.065	.402

CES-D: Centre for Epidemiological studies-Depression Scale; JTCI: The Junior Temperament and Character Inventory; NS: Novelty Seeking; HA: Harm Avoidance; RD: Reward Dependence; P: Persistence; SD: Self-Directedness; CO: Cooperativeness; ST: Self-Transcendence. For age, gender, and the evaluation of the Δ*F* statistic: ***P ≤ .001, and **P < .01, and *P < .05. For the JTCI domains where Bonferroni adjustments were made on the critical *α*, ****P < .0071.

**Table 4:** Hierarchical multiple regression analysis of CES-D scores as a function of JTCI facet scales after controlling for age and gender (*n* = 1236).

Correlates	Standardized beta coefficients											
	Model 1	Model 2	Model 3	Model 4	Model 5	Model 6	Model 7	Model 8	Model 9	Model 10	Model 11	Model 12
Gender	.254^∗∗∗^	.112^∗∗∗^	.119^∗∗∗^	.087^∗∗∗^	.094^∗∗∗^	.115^∗∗∗^	.109^∗∗∗^	.101^∗∗∗^	.112^∗∗∗^	.115^∗∗∗^	.115^∗∗∗^	.115^∗∗∗^
Age	.032	.044^∗^	.021	.025	.024	.023	.018	.015	.020	.020	.020	.014
SD4: self-striving		−.608^∗∗∗^	−.427^∗∗∗∗^	−.310^∗∗∗∗^	−.285^∗∗∗∗^	−.273^∗∗∗∗^	−.272^∗∗∗∗^	−.259^∗∗∗∗^	−.258^∗∗∗∗^	−.241^∗∗∗∗^	−.240^∗∗∗∗^	−.243^∗∗∗∗^
SD2: lack of goal direction			−.306^∗∗∗∗^	−.298^∗∗∗∗^	−.296^∗∗∗∗^	−.285^∗∗∗∗^	−.297^∗∗∗∗^	−.291^∗∗∗∗^	−.283^∗∗∗∗^	−.278^∗∗∗∗^	−.268^∗∗∗∗^	−.277^∗∗∗∗^
HA1: anticipatory worry				.214^∗∗∗∗^	.189^∗∗∗∗^	.202^∗∗∗∗^	.196^∗∗∗∗^	.191^∗∗∗∗^	.193^∗∗∗∗^	.167^∗∗∗∗^	.164^∗∗∗∗^	.157^∗∗∗∗^
ST1: self-forgetfulness					.099^∗∗∗∗^	.092^∗∗∗∗^	.087^∗∗∗∗^	.080^∗∗∗∗^	.068	.061	.068	.066
RD4: independence						−.080^∗∗∗∗^	−.088^∗∗∗∗^	−.089^∗∗∗∗^	−.086^∗∗∗∗^	−.089^∗∗∗∗^	−.084^∗∗∗∗^	−.080^∗∗∗∗^
CO2: empathy							.057	.071	.067	.069	.083^∗∗∗∗^	.061
HA4: fatigability								.065	.065	.061	.056	.063
NS4: disorderliness									.048	.056	.058	.060
HA2 fear of uncertainty										.067	.077	.089
ST2: transpersonal identification											−.055	−.054
SD3: resourcefulness												.057

_Adj_ *R* ^2^	.064	.413	.474	.502	.509	.514	.517	.520	.552	.524	.526	.528
Δ*F*	42.76	732.00	143.05	68.93	19.81	14.41	7.64	8.61	4.91	6.78	6.38	5.71
Δ*R* ^2^	.065	.349	.061	.028	.008	.006	.003	.003	.002	.002	.002	.002

CES-D: Centre for Epidemiological studies-Depression Scale; JTCI: The Junior Temperament and Character Inventory. For age, Gender, and the evaluation of Δ*F* statistics, ***P < .001; **P < .01; *P < .05. For the JTCI facets Bonferroni adjustments were made on the critical *α* level, ****P < .0017.

**Table 5:** Logistic regression predicting the likelihood of using MoodGYM (*n* = 691).

	*B*	SE	Wald	df	*P*	Odds ratio	95% CI for odds ratio	
							Lower	Upper
CES-D	.046	.019	5.67	1	.017^∗^	1.047	1.008	1.087
GSE	−.039	.033	1.39	1	.239	.962	.901	1.026
Gender	.477	.346	1.90	1	.168	1.611	.817	3.175
Novelty seeking	−.019	.022	.780	1	.377	.981	.940	1.024
Harm avoidance	−.047	.025	3.57	1	.059	.954	.909	1.002
Reward dependence	−.048	.018	6.87	1	.009^∗^	.953	.920	.988
Persistence	.014	.025	.33	1	.564	1.015	.966	1.066
Self directedness	.021	.026	.64	1	.425	1.021	.970	1.074
Cooperativeness	.017	.023	.54	1	.461	1.017	.973	1.063
Self-transcendence	.016	.022	.55	1	.460	1.016	.974	1.060
Constant	−.214	2.654	.01	1	.936	.807		

Significant *P* < .05*.

**Table 6:** Logistic regression predicting the likelihood of using MoodGYM (*n* = 691).

	*B*	SE	Wald	df	*P*	odds ratio	95% CI for odds ratio	
							Lower	Upper
CESD	.052	.023	5.01	1	.025^∗^	1.054	1.007	1.103
GSE	−.057	.038	2.22	1	.136	.945	.877	1.018
Gender	.973	.415	5.50	1	.019^∗^	2.646	1.173	5.969
ns1 (exploratory excitability)	.027	.071	.14	1	.704	1.027	.894	1.181
ns2 (impulsiveness)	.000	.072	.00	1	.999	1.000	.869	1.151
ns3 (extravagance)	.037	.067	.31	1	.580	1.038	.911	1.182
ns4 (regimentation)	−.154	.069	4.98	1	.026^∗^	.858	.749	.981
ha1(anticipatory worry)	−.163	.082	3.91	1	.048^∗^	.850	.723	.999
ha2 (fear of uncertainty)	−.017	.068	.06	1	.804	.983	.861	1.123
ha3 (shyness with strangers)	.035	.081	.18	1	.671	1.035	.883	1.214
ha4 (vigor)	−.153	.075	4.22	1	.040^∗^	.858	.741	.993
rd1 (sentimentality)	−.082	.064	1.65	1	.198	.921	.813	1.044
rd2 (openness to warm communication)	.049	.045	1.16	1	.281	1.050	.961	1.147
rd3 (detachment)	−.152	.091	2.80	1	.094	.859	.719	1.026
rd4 (independence)	−.124	.063	3.92	1	.048^∗^	.883	.781	.999
p1 (eagerness to effort)	−.047	.082	.33	1	.563	.954	.812	1.120
p2 (work hardened)	.090	.081	1.26	1	.263	1.094	.935	1.282
p3 (ambitious)	.004	.071	.00	1	.958	1.004	.874	1.153
p4 (perfectionist)	−.230	.111	4.30	1	.038^∗^	.795	.640	.987
sd1 (blaming)	−.034	.076	.20	1	.653	.966	.833	1.121
sd2 (purposefulness)	.003	.089	.00	1	.974	1.003	.842	1.194
sd3 (resourcefulness)	.373	.113	10.87	1	.001^∗∗^	1.453	1.163	1.814
sd4 (self-striving)	−.018	.059	.09	1	.764	.982	.875	1.103
co1 (social acceptance)	.087	.092	.90	1	.342	1.091	.912	1.305
co2 (social disinterest)	−.152	.091	2.77	1	.096	.859	.719	1.027
co3 (helpfulness)	.111	.089	1.56	1	.212	1.117	.938	1.331
co4 (compassion)	.075	.097	.60	1	.438	1.078	.892	1.303
co5 (self-serving advantage)	−.081	.081	.99	1	.320	.922	.786	1.082
st1 (self-forgetfulness)	.011	.065	.03	1	.865	1.011	.890	1.148
st2 (transpersonal identification)	.173	.089	3.79	1	.052	1.188	.999	1.414
st3 (rational materialism)	−.056	.051	1.21	1	.271	.945	.856	1.045
Constant	2.017	3.28	.38	1	.539	7.513	—	—

Significant *P* < .05* and *P* ≤ .001.
